# Autophagy attenuates tubulointerstital fibrosis through regulating transforming growth factor-β and NLRP3 inflammasome signaling pathway

**DOI:** 10.1038/s41419-019-1356-0

**Published:** 2019-01-28

**Authors:** Sun Ah Nam, Wan-Young Kim, Jin Won Kim, Sang Hee Park, Hong Lim Kim, Myung-Shik Lee, Masaaki Komatsu, Hunjoo Ha, Ji Hee Lim, Cheol Whee Park, Chul Woo Yang, Jin Kim, Yong Kyun Kim

**Affiliations:** 1 Cell Death Disease Research Center, College of Medicine, The Catholic University of Korea, Seoul, Korea; 2 Institute of Clinical Medicine Research of Bucheon St. Mary’s Hospital, Bucheon-si, Korea; 3 Integrative Research Support Center, College of Medicine, The Catholic University of Korea, Seoul, Korea; 4 Severans Biomedical Research Institute and Department of Internal Medicine, Yonsei University College of Medicine, Seoul, Korea; 5 Department of Biochemistry, Niigata University School of Medicine, Niigata, Japan; 6 Graduate School of Pharmaceutical Sciences, College of Pharmacy, Ewha Womans University, Seoul, Republic of Korea; 7 Department of Internal Medicine, College of Medicine, The Catholic University of Korea, Seoul, Korea

## Abstract

Renal fibrosis is the final common pathway of various renal injuries and it leads to chronic kidney disease. Autophagy is a cellular process of degradation of damaged cytoplasmic components and regulates cell death and proliferation. Cellular response during unilateral ureteral obstruction (UUO) is tubular segment specific. Thus the role of autophagy on renal tubulointerstitial fibrosis (TIF) after UUO may be different according to segment of nephron. The role of autophagy during UUO remains unclear especially in distal tubules. In this study, we investigated the role of autophagy in distal tubules on renal TIF using conditional knockout mice in which Atg7 was genetically ablated specifically in distal tubular epithelial cell (TEC). In green fluorescent protein (GFP)-LC3 transgenic mice, GFP-LC3 puncta was highly expressed in distal tubular cells of the obstructed kidneys after UUO. Genetic deletion of Atg7 specifically in distal TEC increased renal tubulointerstial fibrosis and epithelial-mesenchymal transition-like phenotype change after UUO through Smad4-dependent transforming growth factor (TGF)*-*β pathway. Distal tubule-specific autophagy-deficient mice increased the accumulation of damaged mitochondria and SQSTM1/p62-positive aggregates in the obstructed kidney and resulted in increased expression of NLRP3 inflammasome, interleukin (IL) 1-β and caspase-1. Distal TEC-specific Atg7 deletion enhanced apoptosis of TECs after UUO. In summary, our data showed that autophagy in distal TEC plays a protective role in development of renal tubulointerstial fibrosis through regulating the expression of TGF-β an IL1-β after UUO.

## Introduction

Renal fibrosis is the final common pathway of various renal injuries and leads to chronic kidney disease and end-stage renal disease^1,2^. Renal fibrosis is characterized by excessive production and progressive accumulation of extracellular matrix (ECM) protein, such as collagen I and fibronectin^1^. The matrix-producing fibroblasts in the renal interstitium are considered to be the main source of increased ECM protein during renal fibrosis^3,4^. Renal tubular epithelial cells contribute to the pathogenesis of renal fibrosis by modulating their apoptosis and proliferation, or by secretion of cytokines inducing inflammation and the formation of fibroblast in response to various injuries^1^.

Autophagy is an evolutionarily conserved, lysosomal-mediated cellular process of degradation of damaged organelles, protein aggregates, and other macromolecules in the cytoplasm and regulates cell death under normal physiological conditions as well as pathological conditions^5,6^. Autophagy is involved in renal diseases, including acute kidney injury, glomerular diseases, and aging of kidney^7–9^.

Autophagy has been reported to regulate renal fibrosis but its role on renal fibrosis remains unclear^10–13^. We previously reported that autophagy has a protective role in renal fibrosis induced by Unilateral ureteral obstruction (UUO)^10^. A previous study demonstrated that autophagy regulates the expression of transforming growth factor (TGF)-β and suppress renal tubulointerstial fibrosis in UUO model using LC3^−/−^ mice and beclin 1 heterozygous mice^11^. In contrast, another study reported that the persistent activation of autophagy promotes renal tubulointerstitial fibrosis (TIF) during UUO in proximal tubular cell-specific Atg7 knockout mice^9^. This discrepancy is may be due to the different role of autophagy for TIF according to the different cell types or the diverse cross talk among the cells^14^. In addition, cellular response after UUO differs between proximal and distal nephron^15^. While the distal nephron is an important component in UUO injury, the mechanism of distal nephron injury after UUO still remains unclear.

NLRP3 (NOD-like receptor, pyrin domain-containing 3) is a member of the NOD-like receptors (NLRs) involving innate immune response^16^. NLRP3 forms a protein complex, the inflammasome, which induces caspase-1 activation that results in the maturation and secretion of pro-inflammatory cytokines such as (interleukin) IL-1β and IL-18^17^. Thus, NLRP3 inflammasome signaling pathway regulates a variety of host innate immune defense pathways in response to pathogen or damage-associated molecular patterns by microbial and nonmicrobial stimuli^17^. In renal injury, a previous study demonstrated that absence of NLRP3 attenuated tubular injury, inflammation, and fibrosis after UUO^17^. There is growing evidence that autophagy regulates NLRP3 inflammasome signaling pathway^16^. Thus, we hypothesized that the autophagy may regulate the renal TIF after UUO through the regulation of NLRP3 inflammasome signaling pathway.

In this study, we demonstrated that autophagy deficiency in distal tubular epithelial cells (TECs) resulted in an increase of damaged mitochondria and oxidative stress, which activated NLRP3 inflammasome/caspase-1/IL-1β signaling pathway and induced apoptosis of TECs. These data provide an insight for regulating autophagy as a therapeutic option for CKD.

## Results

### Autophagy is induced in Distal TECs after UUO

**Fig. 1:**
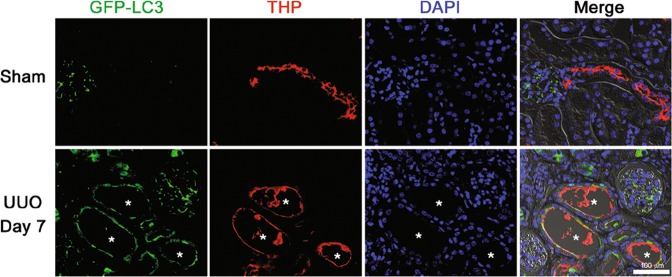
Autophagy is induced in Distal TECs after UUO. Representative immunofluorescent staining of THP (red) in the kidney of GFP-LC3 transgenic mice after sham operation or 7 days after UUO. The asterisk: GFP-LC3 puncta were expressed in the THP-positive tubular cells of obstructed kidneys after UUO. Scale bars, 100 μm

First, to determine the induction of autophagy in renal distal tubular epithelial cells after UUO, we used green fluorescent protein (GFP)-LC3 transgenic mice. In sham operation, GFP-LC3 puncta were rarely observed in renal TECs. After UUO, the expression of GFP-LC3 puncta was increased and GFP-LC3 puncta were co-localized with Tamm–Horsfall protein (THP)-positive cells (Fig. 1). Western blot analysis revealed that LC3-II/LC3-I was significantly increased after UUO in wild-type (WT) mice (Supplementary Fig. 1a). These data suggest the induction of autophagy in renal distal TECs after UUO.

### Distal TEC-specific Atg7 deletion enhances tubulointerstitial fibrosis after UUO

**Fig. 2:**
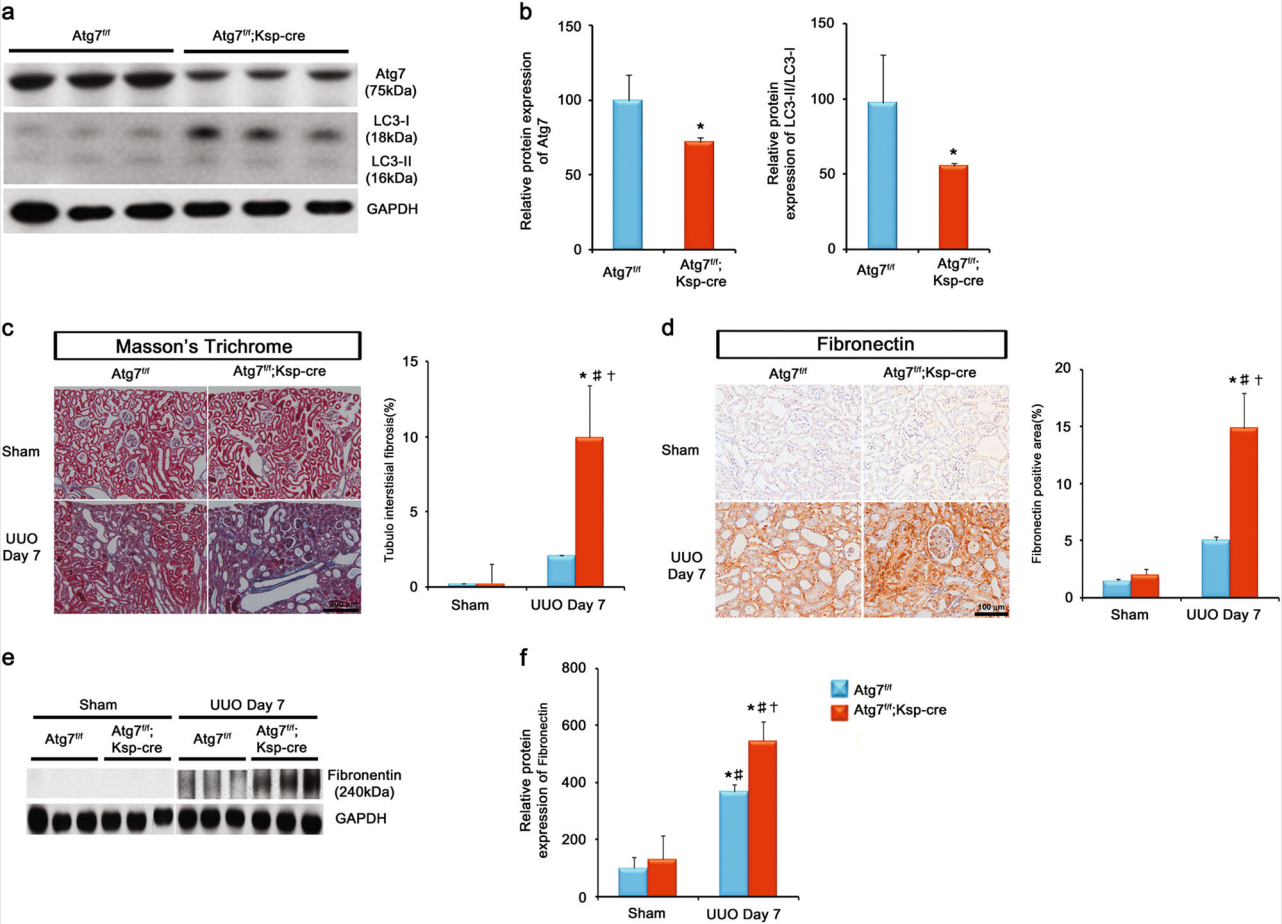
Distal TEC-specific Atg7 deletion enhances tubulointerstitial fibrosis after UUO. **a** Representative immunoblot analysis and densitometry of Atg7 and LC3. **b** Quantification of Atg7 protein and LC3-II/LC3-I. **c** Masson’s trichrome staining from WT and tubular epithelial cell-specific Atg7 KO mice 7 days after UUO, showing increased extracellular matrix deposition within the tubulointerstitium in the distal TEC-specific Atg7 KO mice 7 days after UUO. Scale bars, 100 μm. **d** Immunohistochemical staining of fibronectin expression from WT and TEC-specific Atg7 KO mice 7 days after UUO. Increased expression of fibronectin was evident in the obstructed kidneys of tubular epithelial cell-specific Atg7 KO mice. Scale bars, 100 μm. **e** The protein expression of fibronectin was examined by immunoblot from mouse kidney lysates. **f** Quantification of fibronectin protein (*n* = 5, densitometry; *P < 0.01 versus kidney of WT mice with sham operation; †*P* < 0.01 versus kidney of tubular epithelial cell-specific Atg7 KO mice with sham operation; ‡*P* < 0.01 versus obstructed kidney of WT mice 7 days after UUO)

To investigate the functional role of autophagy-induced renal distal TECs in the obstructed kidney after UUO on renal TIF, we generated conditional knockout mice in which Atg7 is genetically ablated specifically in distal TECs (Atg7^*flox/flox*^;Ksp-Cre^+^). The protein expression of Atg7 from western blot analyses of whole-kidney lysates was significantly decreased in kidneys of Atg7^*flox/flox*^;Ksp-Cre^+^ mice compared with those of WT mice (Fig. 2a and b). LC3-II/LC3-I was significantly decreased in Atg7^*flox/flox*^;Ksp-Cre^+^ mice (Fig. 2a and b). These data confirm an efficient deletion of Atg7 in TECs. No obvious histologic phenotype was not observed in Atg7^*flox/flox*^;Ksp-Cre^+^ mice (Supplementary Fig. 1b).

We next examined the effect of distal TEC-specific deletion of Atg7 in renal TIF induced by UUO. Masson’s trichrome staining revealed that increased extracellular matrix deposition within the tubulointerstitium at 7 days after UUO in WT mice compared with sham-operated kidneys, which was substantially increased in the obstructed kidneys of Atg7^*flox/flox*^;Ksp-Cre^+^ mice (Fig. 2c). Consistently, similar data were obtained by immunohistochemical staining and the western blot analyses for fibronectin (Fig. 2d–f). Plasminogen activator inhibitor 1 (PAI-1) regulates fibrinolysis and the plasmin-mediated ECM matrix metalloproteinase activation.^18,19^ PAI-1 contributes to renal fibrosis by promoting migration of profibrotic cells through a protease-independent mechanism.^19^ In this study, immunohistochemical staining and western blot analyses revealed upregulation of PAI-1 in obstructed kidneys of Atg7^*flox/flox*^;Ksp-Cre^+^ mice after UUO (Supplementary Fig. 1c and d).

Taken together, these data indicate that distal TEC-specific Atg7 deletion increases ECM protein and enhances renal TIF after UUO.

### Distal TEC-specific Atg7 deletion activates TGF-β/Smad4 signaling and enhances tubular EMT-like phenotype change after UUO

**Fig. 3:**
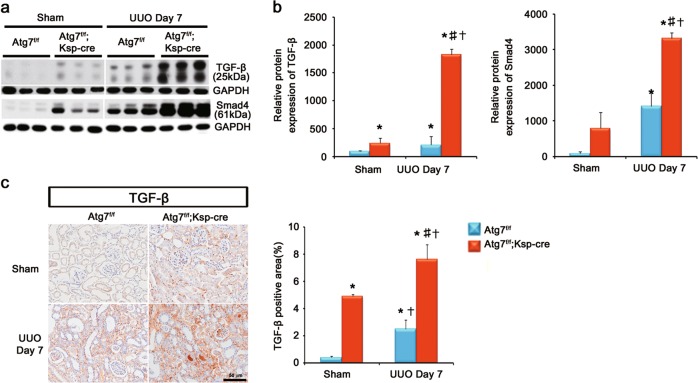
Distal TEC-specific Atg7 deletion activates TGF-β/Smad4 signaling after UUO. **a** Representative immunoblots and **b** densitometry of expression of TGF-β and Smad4. **c** Immunostaining of expression of TGF-β, showing increased TGF-β in the obstructed distal TEC-specific Atg7 KO mice. Scale bars, 50 μm. (*n* = 5, densitometry; *P < 0.01 versus kidney of WT mice with sham operation; †*P* < 0.01 versus kidney of tubular epithelial cell-specific Atg7 KO mice with sham operation; ‡*P* < 0.01 versus obstructed kidney of WT mice 7 days after UUO)

TGF-β is a major cytokine mediating renal TIF by inducing the production of ECM proteins and may be regulated by autophagy degradation^11,20^. TGF-β/Smad signaling is a major pathway leading to renal TIF^20^. Thus, we investigated the effect of distal TEC-specific Atg7 deletion on the expression of the TGF-β/Smad pathway after UUO. The protein expression of TGF-β and Smad4 markedly increased in obstructed kidneys of Atg7^*flox/flox*^;Ksp-Cre^+^ mice compared with those of WT mice, as demonstrated by western blot analyses (Fig. 3a and b). Immunohistochemical staining revealed upregulation of TGF-β in the interstitium of the obstructed kidneys of Atg7^*flox/flox*^;Ksp-Cre^+^ mice after UUO (Fig. 3c).

**Fig. 4:**
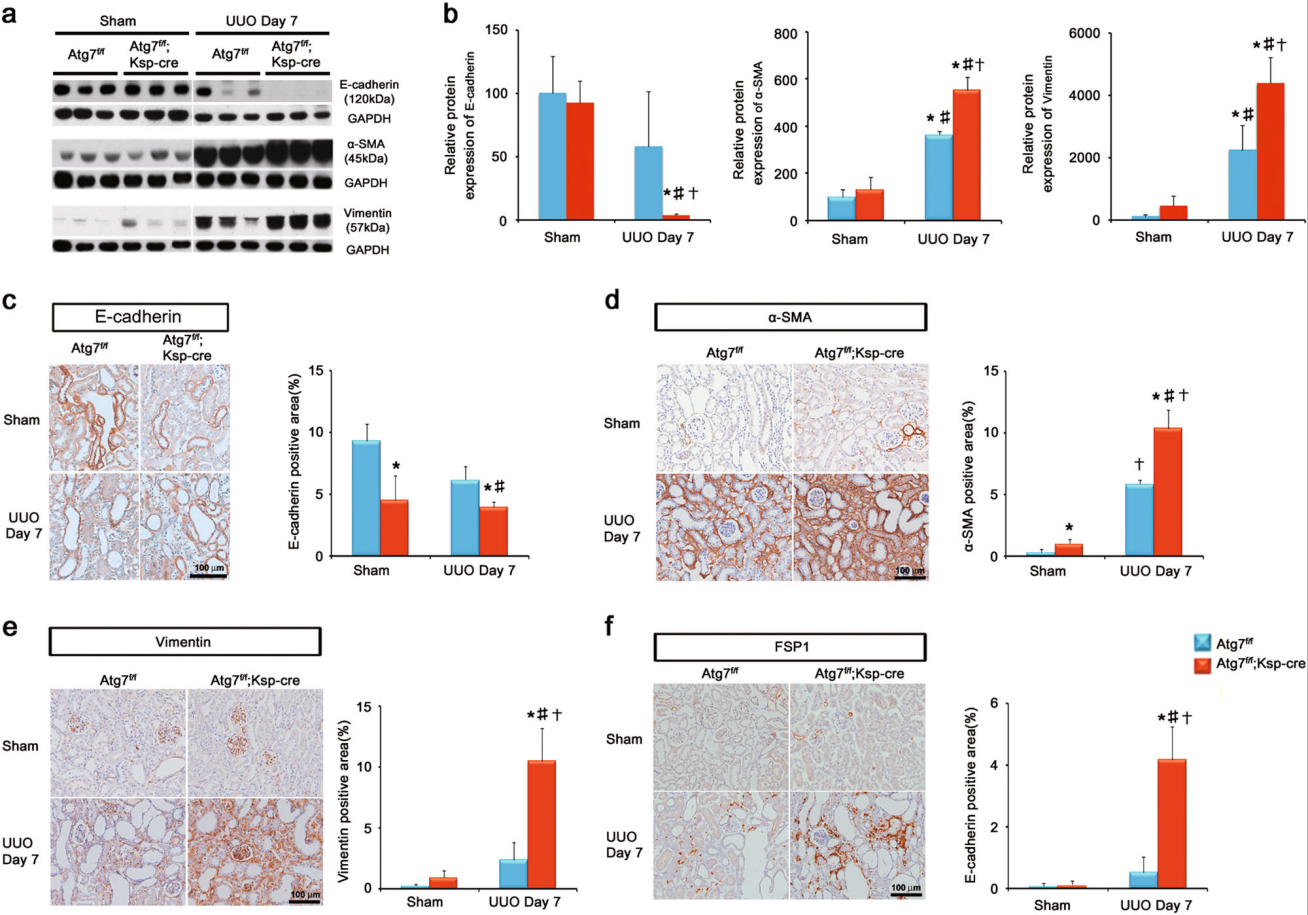
Distal TEC-specific Atg7 deletion enhances tubular EMT-like phenotype and the accumulation of interstitial myofibroblasts change after UUO. **a** Representative immunoblots and **b** densitometry of expression of E-cadherin, α-SMA and vimentin. **c** Immunostaining and quantification of E-cadherin. Scale bars, 100 μm. **d** Immunostaining and quantification of α-SMA. Scale bars, 100 μm. **e** Immunostaining and quantification of and vimentin. Scale bars, 100 μm. **f** Immunostaining and quantification of FSP-1 Scale bars, 100 μm. (*n* = 5, densitometry; *P < 0.01 versus kidney of WT mice with sham operation; †*P* < 0.01 versus kidney of tubular epithelial cell-specific Atg7 KO mice with sham operation; ‡*P* < 0.01 versus obstructed kidney of WT mice 7 days after UUO)

TGF-β may induce epithelial to mesenchymal transition (EMT)-like phenotype changes during the development of renal fibrosis^20^. Thus, we examined the phenotype markers of EMT including E-cadherin, α-smooth muscle antibody (SMA), vimentin and fibroblast-specific protein (FSP)-1 as a marker of fibroblasts. E-cadherin is an epithelial cell marker and loss of E-cadherin represents the earliest step during TGF-β-induced EMT-like phenotype changes^21^. Western blot analyses showed that the protein expression of E-cadherin was decreased in WT mice at day 7 after UUO, which was substantially decreased in Atg7^*flox/flox*^;Ksp-Cre^+^ mice (Fig. 4a and b). The expression of α- SMA, which is an important marker of myofibroblast^21^, and vimentin, which is a cytoskeleton protein and a specific marker for mesenchymal cells, markedly upregulated in obstructed kidneys of Atg7^*flox/flox*^;Ksp-Cre^+^ mice compared with obstructed kidneys of WT mice after UUO (Fig. 4a and b). Consistently, immunohistochemical staining revealed similar data (Fig. 4c–e). FSP-1-positive cells were substantially increased in the renal interstitium in Atg7^*flox/flox*^;Ksp-Cre^+^ mice compared to those of WT mice after UUO (Fig. 4f). These data indicate that autophagy regulates Smad4-dependent TGF-β pathway and mediates TGF-β-induced tubular EMT-like phenotype changes and the accumulation of interstitial myofibroblasts after UUO.

### Distal TEC-specific Atg7 deletion resulted in the accumulation of p62/SQSTM1 and damaged mitochondria through the oxidative DNA damage

**Fig. 5:**
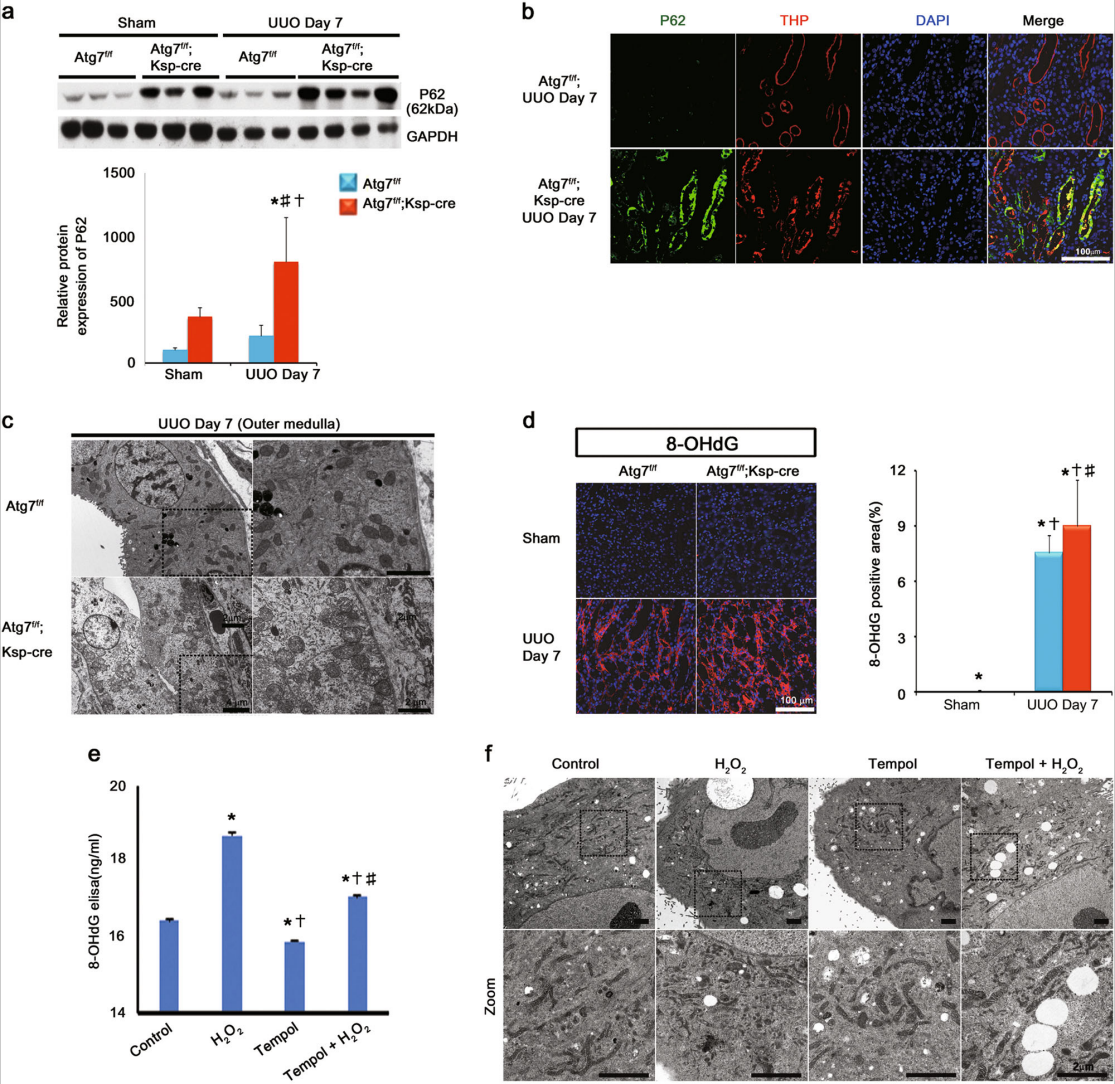
Distal TEC-specific Atg7 deletion resulted in the accumulation of p62/SQSTM1 and damaged mitochondria and increased oxidative stress. **a** Representative immunoblots and **b** densitometry of expression of p62. **b** Representative immunofluorescent staining of THP (red) and p62 (green). The accumulation of p62/SQSTM1 was abundantly increased in the obstructed kidneys of Atg7^*flox/flox*^;Ksp-Cre^+^ mice, which was co-localized with THP which is a targeted-autophagy-deficient tubular cells of Atg7^*flox/flox*^;Ksp-Cre^+^ mice. Scale bars, 50 μm. **c** Ultrastructural alterations in tubular epithelial cell-specific Atg7 deletion after UUO. Majority of mitochondria in tubular epithelial cells had elongated cylindrical shape with organized cristae in WT mice after UUO, whereas the accumulation of damaged mitochondria with spherical shape and cristolysis were observed in Atg7 KO mice. Scale bars, 2 μm. **d** Representative Immunostaining and quantification of expression of 8-OHdG. Scale bars, 100 μm. **e** 8-OHdG levels in MDCK cells treated with H_2_O_2_ with or without Tempol. **f** Ultrastructural changes in MDCK cells induced by H_2_O_2_ with or without Tempol using transmission electron microscopy. Scale bars, 2 μm

p62/SQSTM1 is an ubiquitin-binding scaffold protein that is degraded by autophagy^22^. In previous studies, p62/SQSTM1-associated protein aggregates accumulates in Atg7 autophagy-deficient mouse liver and targeted deletion of p62/SQSTM1 prevents the accumulation of these protein aggregates^23,24^. In this way the levels of p62/SQSTM1 accumulation serves as a good measure of defects in selective autophagy^23,24^. In this study, the accumulation of p62/SQSTM1 was rarely observed at 7 days after UUO in WT mice, which was abundantly increased in the obstructed kidneys of Atg7^*flox/flox*^;Ksp-Cre^+^ mice (Fig. 5a). Furthermore, p62/SQSTM1 was co-localized with THP which is a targeted-autophagy-deficient tubular cells of Atg7^*flox/flox*^;Ksp-Cre^+^ mice (Fig. 5b). These finding indicates a selective and an efficient inhibition of autophagy in distal tubular cells of Atg7^*flox/flox*^;Ksp-Cre^+^ mice.

We next investigated the effect of autophagy deletion on ultrastructural mitochondrial alteration in renal tubular cells after UUO. EM showed the accumulation of damaged mitochondria with spherical shape and cristolysis and lipid inclusions were abundantly observed in outer medulla of kidneys of Atg7^*flox/flox*^;Ksp-Cre^+^ mice (Fig. 5c). Mitochondria are the main source of reactive oxygen species (ROS) generation and mitochondrial DNA also be a main target of ROS^25,26^. In this study, the increased immunoreactivity of 8-hydroxy-2’-deoxyguanosine (8-OHdG), a marker of oxidative DNA damage, was observed in Atg7^*flox/flox*^;Ksp-Cre^+^ mice after UUO (Fig. 5d).

To determine the interaction between oxidative DNA damage and the accumulation of the mitochondria, we treated Madin-Darby Canine Kidney (MDCK) cells with hydrogen peroxide (H_2_O_2_) in vitro and examined the levels of 8-OHdG. H_2_O_2_ treatment of confluent cells increased the level of 8-OHdG, which was prevented by Tempol (an antioxidant as a superoxide dismutase mimetic agent) treatment (Fig. 5e). TEM analyses showed the accumulation of damaged mitochondria with fragmented and spherical shape and cristolysis were increased by H_2_O_2_ treatment, which was recovered by Tempol treatment (Fig. 5f). These findings indicate that the oxidative DNA damage results in the accumulation of damaged mitochondria.

Taken together, our data suggest that autophagy deficiency accumulates the damaged mitochondria through the exacerbation of the oxidative DNA damage after UUO.

### Distal TEC-specific Atg7 deletion activated NLRP3 inflammasome signaling pathway and induced apoptosis of TECs after UUO

**Fig. 6:**
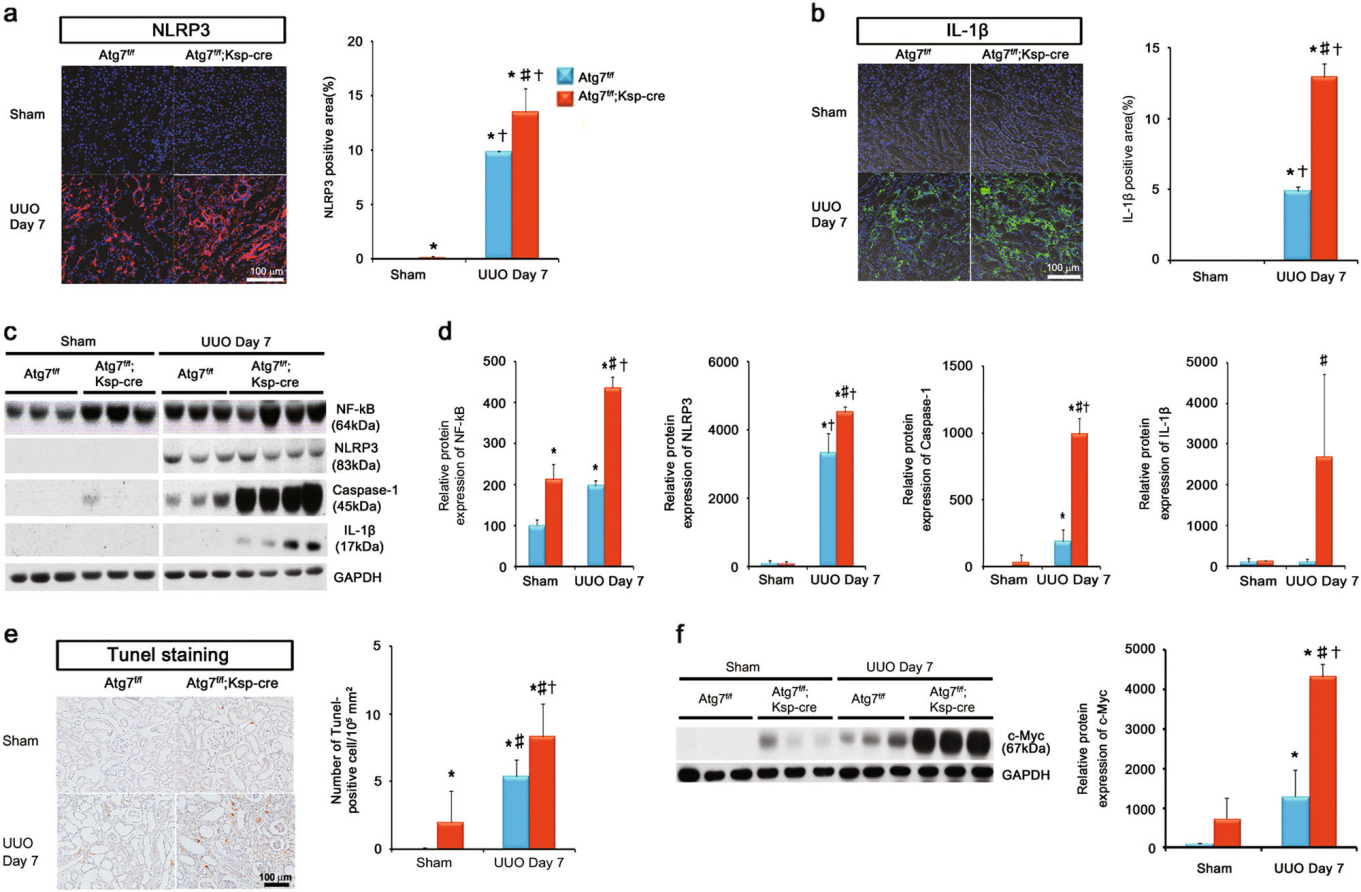
Distal TEC-specific Atg7 deletion activated NLRP3 inflammasome signaling pathway and induced apoptosis of TECs after UUO. **a** Representative immunofluorescent staining and quantification of NLRP3 (red). Scale bars, 100 μm. **b** Representative immunofluorescent staining and quantification of IL-1β (green). Scale bars, 100 μm. **c** Representative immunoblots and **d** densitometry of expression of NF-kB, NLRP3, Caspae-1 and IL-1β. **e** Representative images of TUNEL staining and quantification of TUNEL-positive cells. TUNEL assay revealed that the number of TUNEL-positive cells was significantly greater in obstructed kidneys of tubular epithelial cell-specific Atg7 KO mice compared with WT mice after UUO. Scale bars, 100 μm. **f** Representative immunoblots and densitometry for expression of c-Myc. The protein expression of c-Myc was significantly greater in obstructed kidneys of tubular epithelial cell-specific Atg7 KO mice compared with obstructed kidneys of WT mice after UUO (*n* = 5, densitometry; *P < 0.01 versus kidney of WT mice with sham operation; †*P* < 0.01 versus kidney of tubular epithelial cell-specific Atg7 KO mice with sham operation; ‡*P* < 0.01 versus obstructed kidney of WT mice 7 days after UUO)

The accumulation of damaged mitochondria can induce autophagy as well as inflammasome signaling pathway in innate immunity^16^. In this study, we investigated the effect of autophagy deficiency on NLRP3 inflammasome signaling pathway. Immunohistochemical staining revealed the ablation of Atg7 in distal TEC resulted in the activation of NLRP3 inflammasome and its downstream, IL-1β, after UUO (Fig. 6a and b). Immunoblot assay revealed that Atg7 deficiency in distal TEC induced NF-kB/NLRP3/Caspae-1/ IL-1β signaling pathway after UUO (Fig. 6c and d).

**Fig. 7:**
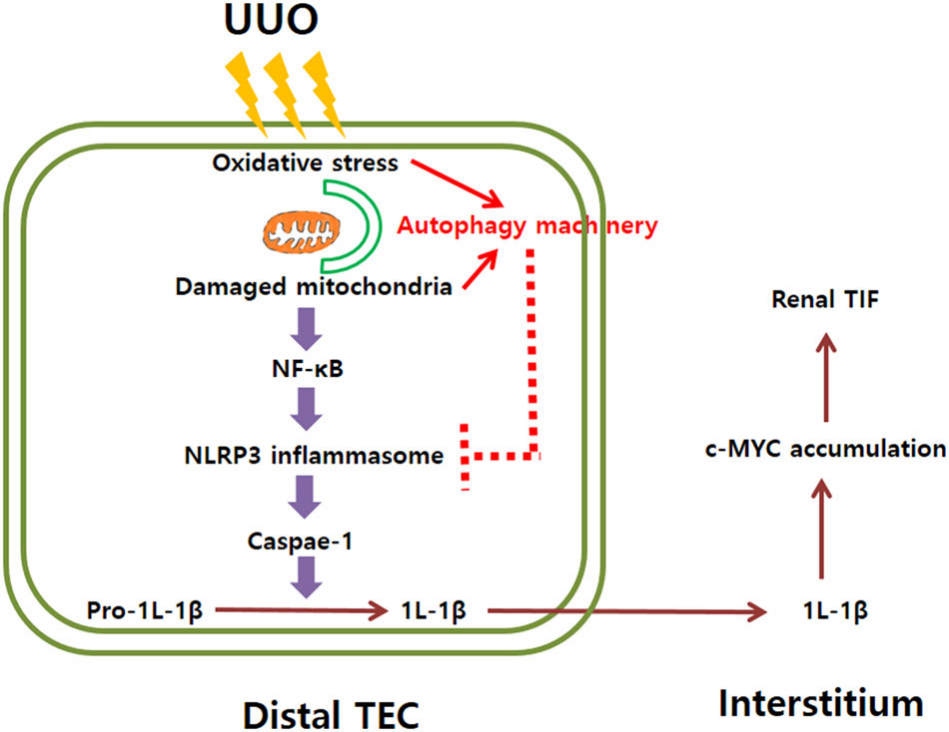
Schematic representation of the potential mechanism of autophagy regulating NLRP3 inflammasome signaling pathway in renal tubulointerstitial fibrosis. Oxidative stress from UUO injury resulted in the accumulation of the damaged mitochondria, which can induce autophagy machinery as well as NLRP3 inflammasome signaling pathway. Activated NLRP3 forms a protein complex, the inflammasome, which induces caspase-1 activation that results in the maturation and secretion of pro-inflammatory cytokines IL-1β. Increased IL-1β stimulates the accumulation of MYC and upregulated MYC target gene, which contributes renal progressive TIF. Induced autophagy resulted from oxidative stress and the accumulation of the damaged mitochondria regulates NLRP3 inflammasome signaling pathway, which attenuates the apoptosis of TECs and renal progressive TIF in response to UUO injury

NLRP3 inflammasome signaling pathway in the context of autophagy regulates cellular apoptosis in response to various forms of cellular injury^10–12,16,25,27^. Thus, we investigated the effect of autophagy on apoptosis of renal TECs after UUO. Terminal deoxynucleotidyl transferase dUTP nick end labeling (TUNEL) assay revealed that the number of TUNEL-positive cells was significantly greater in obstructed kidneys of distal TEC-specific autophagy-deficient mice compared with WT mice after UUO (Fig. 6e). These findings indicated a protective role of autophagy in apoptosis of renal TECs after UUO. Taken together, these data suggest that autophagy in distal TECs regulates apoptosis of renal TEC through the NLRP3/Caspase-1/IL-1β signaling pathway in response to UUO. A recent study demonstrated that the accumulation of MYC and upregulated MYC target gene resulted from IL-1β stimulation was necessary for renal progressive TIF^28^. In this study, the protein expression of c-MYC was substantially increased in the distal TEC-specific autophagy-deficient mice after UUO. These findings suggested that upregulated NLRP3/Caspase-1/IL-1β signaling pathway in autophagy-deficient mice after UUO resulted in the accumulation of c-MYC, which may enhance renal TIF and tubular injury (Fig. 7)

## Materials and methods

### Animals

Atg7^*flox/flox*^ mice were crossed with Ksp-Cre mice (Jackson Laboratories, West Grove, PA, USA) to generate distal tubule-specific Atg7 knockout mice (Atg7^*flox/flox*^;Ksp-Cre mice). Atg7^*flox/flox*^ litermates served as controls. All mice were crossed on a C57BL6 background and only male mice were used in the study. UUO was performed as described previously^11^. Briefly, mice were anesthetized with zoletil and the left ureter was exposed via a left dorsal incision. The mid-ureter was then obstructed using a two-point ligation with silk sutures. The sham-operated mice underwent the same procedure with the exception of obstruction of the left ureter and used as controls. Mice were killed at 3, 7, and 14 days after UUO. The kidneys were briefly perfused with phosphate-buffered saline (PBS, pH 7.4) to rinse away any remaining blood. This was followed by perfusion with periodate-lysine-2% paraformaldehyde solution for 10 min. After perfusion, the kidneys were removed and cut into 1–2 mm thick slices, which were further fixed by immersion in the same fixative overnight at 4 °C. After fixation, the kidney slices were rinsed in PBS and dehydrated in a graded series of ethanol solutions and embedded in paraffin. All the experimental procedures were performed according to the animal care and ethics legislation and the study was approved by the Animal Care Committee of Bucheon Saint Mary’s Hospital.

### Cell culture

Madin-Darby canine kidney cells (MDCK, American Type Culture Collection) were cultured in MEM with 10% FBS (Mediatech Inc.) with streptomycin/penicillin. After becoming confluent, cells were treated with 0.01% hydrogen peroxide (H_2_O_2_) (Sigma, St. Louis, MO) with or without Tempol 0.5 mM for 1 h.

### 8-hydroxy-2′-deoxyguanosine (8-OHdG) assay

The MDCK cells were seeded in 6-well plates at 2 × 10^5^ cells/well. The supernatant of the culture medium and cytoplasmic fraction was collected following exposure to H_2_O_2_ and/or Tempol for 1 h. To determine the occurence of oxidative DNA damage, the OxiSelect™ Oxidative DNA Damage ELISA kit (Cell Biolabs, Inc., San Diego, CA, USA) was used for the detection and quantification of 8-OHdG.

### Antibodies

The antibodies used in this study were as follows: Atg7 (Sigma-Aldrich, St. Louis, MO, USA), LC3B (anti-LC3B; Sigma-Aldrich, St. Louis, MO, USA), P62 (PROGEN Biotechnik GmbH, Heidelberg, Germany), fibronectin (DAKO, Glostrupp, Denmark), TGF-β (R&D systems, Minneapolis, Minnesota, USA), E-cadherin (BD Transduction Laboratories, Lexington, KY, USA), α-SMA (Sigma-Aldrich, St. Louis, MO, USA), vimentin (Santa Cruz Biotechnoligy, California, USA), PAI-1 (Santa Cruz Biotechnoligy, California, USA), NLRP3 (Adipogen, San Diego, USA), aspase-1 (Santa Cruz Biotechnoligy), FSP1 (Thermo scientific, Fremont, USA), IL-β (Cell signaling technology, Inc. Danvers, MA, USA), NF-KB (Abcam, Cambridge, UK), 8-OHdG (JaICA, haruoka, Fukuroi, Shiizuoka, Japan) and GAPDH (Santa Cruz Biotechnology) were used. Apoptosis was detected using an ApopTag Peroxidase In Situ Apoptosis Detection Kit (Millipore, Billerica, MA, USA).

### Immunohistochemical analysis

Some kidney sections were processed and stained with periodic acid-Schiff (PAS) or Masson’s trichrome stain. Other sections were processed for post-embedding immunohistochemistry analysis. After deparaffin, the sections were hydrated and incubated with 0.5% Triton X-100/PBS solution for 30 min and then they were washed with PBS three times. The non-specific binding sites were blocked with normal donkey serum diluted 1:10 in PBS for 1 h, and then the sections were incubated for overnight at 4 °C in a primary antibody. After rinsing in PBS, the sections were incubated in peroxidase-conjugated anti-mouse or anti-rabbit IgG (Jackson ImmunoResearch Laboratories, West Grove, PA) for 1 h. For coloration, the sections were incubated with a mixture of 0.05% 3,3’-diaminobenzidine that contained 0.01% H_2_O_2_ at room temperature until a brown color was visible and they were then washed with Tris buffer (pH 7.6), counterstained with hematoxylin and observed under light microscopy. The sections were scanned and automatically digitized using a (Leica SCN400), and then they were analyzed using the software (Tissuemorph/DP, Visiopharm, Denmark).

### Western blot analysis

The kidney was homogenized in boiling lysis buffer (1% SDS, 1 mM sodium orthovanadate, and 10 mM Tris, pH 7.4) and the protein concentration was determined with the BCA Protein assay kit (Pierce Biotechnology Inc., Rockford, IL, USA). Equal amounts of the protein were separated on SDS–polyacrylamide gel. The gel was transferred onto a NC membrane. For immunodetection, the blots were incubated overnight in PBS that containing 0.1% Tween-20 and 5% skim milk with the primary antibody. The blots were washed and then incubated with a secondary antibody conjugated to horseradish peroxidase (Jackson Immuno Research Laboratories) and the blots were visualized using a western blotting luminol reagent kit (Santa Cruz Biotechnology, Santa Cruz, CA.).

### Electron microscopic (EM) analysis

For observing the autophagy and ultrastructural changes of mithochondria, we performed a conventional transmission EM study. Kidney block samples and MDCK cells were fixed in 2% paraformaldehdyde and 2.5% glutaraldehyde in 0.1 M phosphate buffer for overnight at 4 °C. After washing in 0.1 M phosphate buffer, the samples were postfixed with 1% osmium tetroxide in the same buffer for 1 h at 4 °C. Next the samples were dehydrated with a series of the graded ethyl alcohol solution, exchanged through acetone, and the samples were next embedded in Epon 812.

Ultrathin sections (70~80 nm) were obtained by an ultramicrotome (Leica Ultracut UCT, Germany). Ultrathin sections were double stained with uranyl acetate and lead citrate and they were examined in a transmission electron microscope (JEM 1010, Japan) at 60 kV. For the quantitative determination, 20 field of low magnification (×6000) were randomly selected from each section of the cortex and the amount of autophagosomes per 100 μm^2^ was evaluated.

### Statistics

Values are presented as the mean ± SD. Data were compared between groups using an Mann–Whitney test or Kruskal–Wallis test as appropriate. *P*-values less than 0.05 were considered significant. All statistical analyses were performed using SPSS 16.0 software (Chicago, IL, USA).

## Discussion

In this study, we demonstrated the role and its mechanisms of autophagy in renal tubulointerstitial fibrosis. We showed that genetic deficiency of Atg7 in a distal TECs-specific fashion upregulated the expression of TGF-β/Smad4 signaling pathway, which in turn induced EMT-like phenotype changes and led to accelerated renal tubulointerstitial fibrosis after UUO. We also showed the Atg7 deficiency in distal TECs-induced apoptosis through activating NLRP3 inflammasome signaling pathway after UUO. Our results established that autophagy in distal TECs plays a pivotal role in development of renal tubulointerstitial fibrosis and induction of apoptosis in TECs.

Previous studies investigated the mechanisms of autophagy in development of renal tubulointerstial fibrosis. Using LC3−/− mice or Beclin1 haploinsufficienct mice Ding et al. showed that autophagy deficiency resulted in increased levels of mature TGF-β and was associated with more severe fibrosis and renal tubular epithelial cell apoptosis in the obstructed kidney after UUO^11^. They suggest the induction of autophagy by TGF-β itself promotes mature TGF-β degradation, which subsequently reduce secretion of TGF-β and attenuates renal interstitial fibrosis^11^. In addition, using distal TECs-specific Atg7 knockout mice, we suggest that autophagy machinery regulates the Smad-dependent TGF-β signaling pathway and subsequent EMT-like phenotype changes in renal tubulointerstitial fibrosis.

Myofibroblast has been recognized the main effector cell producing ECM protein during renal fibrosis^1^. The precise origin of myofibroblast is still remains controversial^1^. EMT has been considered to contribute to the myofibroblast pool during renal fibrosis, but recent fate tracing studies revealed the limited contribution on the myofibroblast pool^21,29,30^. Furthermore, in vivo studies, EMT was not observed mice with overexpression of TGF-β1 in renal tubules or autophagy-deficient mice, such as LC3−/− mice or Beclin1 haploinsufficienct mice^11,30^. Thus, autophagy may be unlikely to induce EMT during renal fibrosis. Unexpectedly, this study showed the EMT-like phenotype changes in distal TECs-specific Atg7 knockout mice after UUO. We cautiously suggest that autophagy may regulate renal fibrosis through EMT-like phenotype changes or partial EMT although its contribution to renal fibrosis may be limited. Nevertheless, the direct evidences with cell fate tracing are still needed to clarify the contribution of autophagy in EMT during renal fibrosis.

One of the interesting points of this study is the role of autophagy in activation of NLRP3 inflammasome signaling pathway during renal fibrosis. In innate immunity, autophagy has been reported to play a number of roles in regulating inflammasome activation and IL-1 family cytokine secretion by the removal of inflammasome-activating endogenous signals or the sequestration and degradation of inflammasome components^16^. This role for autophagy in NLRP3 inflammasome signaling pathway is not limited to immune cells. In diabetic retinopathy, a study showed that inhibition of autophagy in retinal pigment epithelial cells induced IL-1β release via ROS mediated NLRP3 inflammasome activation under high glucose condition^16^. Our data suggested that autophagy deficiency in distal TECs resulted in an increase of damaged mitochondria and oxidative stress, which activated NLRP3 inflammasome/caspase-1/IL-1β signaling pathway and induced apoptosis of TECs. Activated IL-1β stimulates the accumulation of MYC and upregulated MYC target gene, which contributes renal progressive TIF. Induced autophagy resulted from oxidative stress and the accumulation of the damaged mitochondria regulates NLRP3 inflammasome signaling pathway, which attenuates the apoptosis of TECs and renal progressive TIF in response to UUO injury (Fig. 7). Our data suggest the linking mechanism of autophagy with renal tubular apoptosis and fibrosis after UUO.

## Conclusions

In conclusion, we have demonstrated that induction of autophagy in distal TECs after UUO has a protective role in renal tubulointerstial fibrosis through the regulation of TGF-β/Smad4 signaling pathway and NLRP3 inflammasome/caspase-1/IL-1β signaling pathway. Thus, precise regulation of autophagy may be a therapeutic option for CKD.

## Supplementary information

Supplementary information

supplemental figure legends
