# Supplementary figures and images for: Autophagy attenuates tubulointerstital fibrosis through regulating transforming growth factor-β and NLRP3 inflammasome signaling pathway
Source: Cell Death Dis. 2019 Jan 28;10(2):78. doi: 10.1038/s41419-019-1356-0 (PMC6349890; doi:10.1038/s41419-019-1356-0)

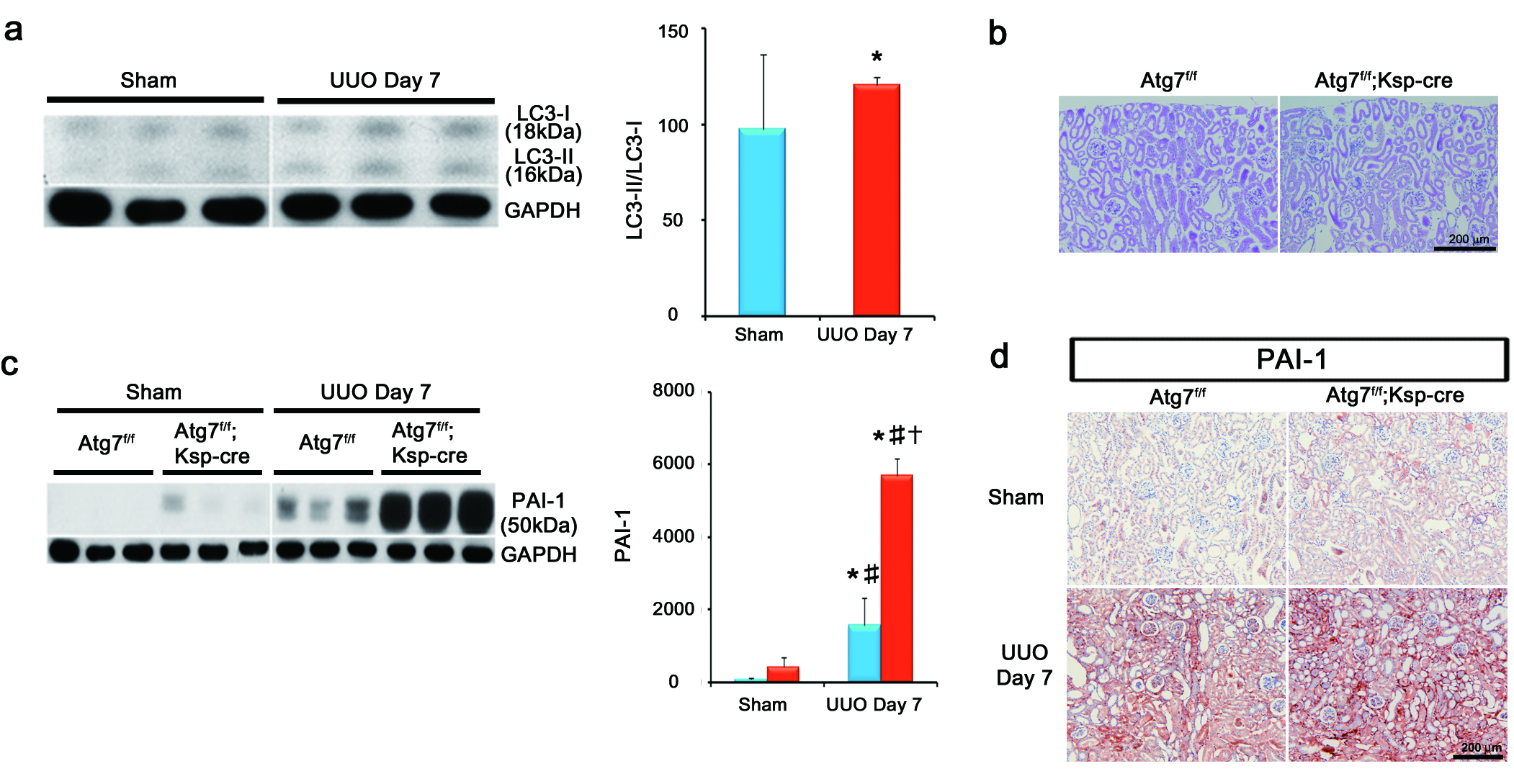

Supplement: Supplementary file 1 — Supplementary information [file 41419_2019_1356_MOESM1_ESM.jpg]
