# Supplementary material for: Autophagy attenuates tubulointerstital fibrosis through regulating transforming growth factor-β and NLRP3 inflammasome signaling pathway
Source: Cell Death Dis. 2019 Jan 28;10(2):78. doi: 10.1038/s41419-019-1356-0 (PMC6349890; doi:10.1038/s41419-019-1356-0)
Supplement: Supplementary file 2 — supplemental figure legends [file 41419_2019_1356_MOESM2_ESM.docx]

**Supplementary information**

**Distal TEC-specific Atg7 deletion increases expression of PAI-1 after UUO.** **a** Representative immunoblots and densitometry of LC3-1 and LC3-II. **b** Representative H&E staining. **c** Representative immunoblots and densitometry for expression of PAI-1. Scale bars, 200 μm. **d** Representative immunostaining for expression of PAI-1, showing upregulation of PAI-1 in obstructed kidneys of tubular epithelial cell-specific Atg7 KO mice after UUO. Scale bars, 200 μm. (n=5, densitometry; ^*^P < 0.01 versus kidney of WT mice with sham operation; ^#^P < 0.01 versus kidney of tubular epithelial cell-specific Atg7 KO mice with sham operation; ^†^P < 0.01 versus obstructed kidney of WT mice 7 days after UUO).
